# A Multifunctional Nanocage-based MOF with Tri- and Tetranuclear Zinc Cluster Secondary Building Units

**DOI:** 10.1038/s41598-018-21382-1

**Published:** 2018-02-15

**Authors:** Zhongyuan Zhou, Xiushuang Xing, Chongbin Tian, Wei Wei, Dejing Li, Falu Hu, Shaowu Du

**Affiliations:** 1 State Key Laboratory of Structural Chemistry, Fujian Institute of Research on the Structure of Matter, Chinese Academy of Sciences, Fuzhou, 350002 P.R. China; 2 University of Chinese Academy of Sciences, Beijing, 100039 P.R. China

## Abstract

A new Zn-cluster based MOF, [Zn_21_(BTC)_11_(μ_3_-OH)_3_(μ_4_-O)_3_(H_2_O)_18_]·21EtOH (**1**) (H_3_BTC = 1,3,5-benzenetricarboxylic acid), with two different types of cluster nodes has been successfully synthesized from Zn^2+^ and H_3_BTC under the solvothermal conditions. Single crystal X-ray diffraction studies reveal that **1** is a 3D trinodal (3,5,6)-c framework which features a large octahedral cage organized by nine Zn_3_O and nine Zn_4_O clusters SBUs and twenty-four triangular BTC^3−^ linkers. The Eu^3+^/Tb^3+^-incorporated derivative of **1** with 0.251% Eu^3+^ and 0.269% Tb^3+^ exhibits tunable luminescence from yellow to white and then to blue-green by changing the excitation wavelength from 308 to 315 nm. Metal ion exchange with Cu^2+^ affords isomorphous Cu-based MOF with enhanced N_2_ and CO_2_ adsorption capacity. In addition, **1** can act as a selective luminescent sensor for Cu^2+^ and Al^3+^ ions.

## Introduction

Over the past two decades, interest in metal−organic frameworks (MOFs) has increased significantly not only because of their intriguing architectures, high crystallinity, exceptional porosity and diverse modularity, but also due to their promising applications in various fields, such as gas adsorption and separation, optical luminescence, catalysis, energy storage and sensing^1–4^. Although a significant number of MOFs have been synthesized and their physical properties have been examined, MOFs are still quite new materials. Hence, the design and synthesis of different kinds of MOFs is necessary to gain more knowledge about their structural diversity and investigate their various properties. While MOF nodes can be composed of single metal ions, they can also be made up of discrete metal-containing clusters, so called secondary building units (SBUs). These metal-cluster SBUs offer an opportunity to design and synthesize highly connected, non-interpenetrating networks with enhanced framework stability and porosity. Among metal cluster SBUs, the Zn based clusters, such as di-, tri-, tetra- and pentanuclear zinc carboxylate clusters are particular useful to build porous networks, since they have a richer variety of size and geometry that allow for more elaborate structural design^5^. Indeed, a plenty of MOFs have been created by assembling Zn based clusters and organic ligands, however, those constructed by two different types of zinc carboxylate clusters, which may further facilitate the structural diversity of Zn-MOFs, are still rare^6,7^.

On the other hand, metal ion exchange is an emerging synthetic route for modifying the secondary building units of MOFs without changing their framework topology. This approach not only can improve the properties of MOF materials, but also allow the preparation of isomorphous MOFs in a single crystal-to-single crystal fashion that cannot be obtained through conventional synthetic routes. Cu^2+^ ion, for example is more likely to replace Zn^2+^ in MOFs. So far, such cation exchanges usually occur at single zinc nodes or paddlewheel zinc carboxylate units^8–10^, those that take place at zinc cluster SBUs are less known. In this work, we demonstrate the replacement of Zn^2+^ by Cu^2+^ at the tri- and tetranuclear zinc clusters in a nanocage-based MOF, resulting in the formation of a Cu analogue with enhanced gas adsorption properties.

Multi-colour emission materials (especially white light) have received increasing attention because they have shown great promise in a variety of applications, from displays, solar cells, to light-emitting diodes. Recently, MOFs have been utilized to generate tunable colour and white light emission through doping appropriate amount of Eu^3+^ and/or Tb^3+^ ions in a single lattice framework composed of Ln^3+^ or non-lanthanide metal ions^11–16^. This approach still remains a great challenge owing to the difficulty of precisely controlling the ratio of different Ln^3+^ ions in one single framework. Another alternative approach to realize colour-tunable luminescence is to incorporate Ln^3+^ species in some microporous luminescence MOFs. However, there are some limitations of these host-gust systems in terms of the judicious selection of suitable host framework and adjusting the incorporation amounts of different Ln^3+^ ions^17–21^.

In the past decade, luminescent MOFs have emerged as promising candidates for the rapid, sensitive and accurate recognition of metal ions^22–27^. The recognition of metal ions plays a very important role in many aspects, including our life^28,29^. The Cu^2+^ and Al^3+^ ions, for instance, are necessary for maintenance of human metabolism. Nevertheless, high concentrations of Cu^2+^ and Al^3+^ can lead to many adverse health effects. Therefore, the design and synthesis of luminescence MOFs capable of sensing Cu^2+^ and Al^3+^ is very important^30,31^. Herein we report a novel Zn-cluster based MOF, [Zn_21_(BTC)_11_(µ_3_-OH)_3_(µ_4_-O)_3_(H_2_O)_18_]·21EtOH (**1**) (H_3_BTC = 1, 3, 5-benzenetricarboxylic acid) built from a triangular Zn cluster SBU, a tetrahedral Zn cluster SBU and a tritopic linker BTC^3−^.Tunable colour and white light emission can be achieved by varying the excitation wavelength and incorporating appropriate amount of Eu^3+^/Tb^3+^ in the pore of **1**. In addition, compound **1** also exhibits a great potential as a luminescence sensing material for Cu^2+^ and Al^3+^ ions.

## Results and Discussion

### Synthesis and description of crystal structure

**Figure 1:**
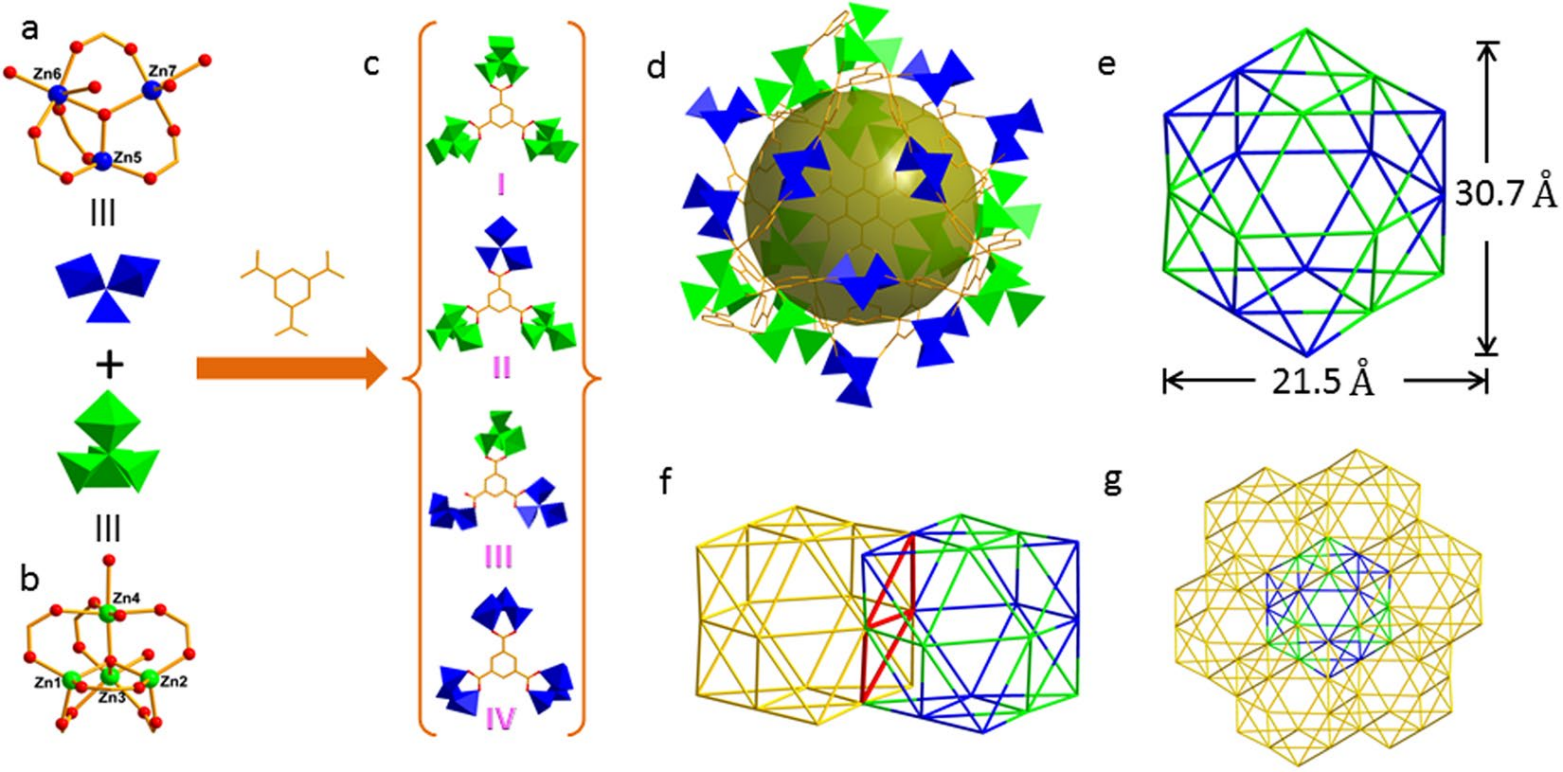
(**a**) The Zn_3_O SBU; (**b**) The Zn_4_O SBU; (**c**) The linking modes of BTC^3−^ ligand; (**d**) View of the octahedral cage constructed by Zn_3_O and Zn_4_O cluster nodes and BTC^3−^ linkers; (**e**) View of the octahedral cage by connecting Zn_3_O and Zn_4_O cluster nodes; (**f**) Two octahedral cages are connected by sharing two edge-fused triangles; (**g**) View of the one octahedral cage surrounded by six identical cages.

Colourless crystals of **1** were synthesized by the solvothermal reaction of Zn(NO_3_)_2_·6H_2_O,H_3_BTC and 4-cyanopyridine in a 1:1:2 molar ratio, in ethanol (10 ml) at 110 °C for three days. Single crystal X-ray diffraction studies reveal that **1** crystallizes in the trigonal space group *R*3. The asymmetric unit of **1** contains seven Zn^2+^ ions, 11/3 BTC^3−^ ligands, one μ_3_-OH^−^ anion, one μ_4_-O_2_^−^ anion and six coordinated water molecules (Fig. S1). The structure contains two types of Zn clusters. One is the trinuclear cluster [Zn_3_(µ_3_-OH)(COO)_5_(H_2_O)_3_] (simplified as Zn_3_O) and the other is the tetranuclear cluster [Zn_4_(µ_4_-O)(COO)_6_(H_2_O)_3_] (simplified as Zn_4_O). In the Zn_3_O cluster, there is a µ_3_-OH group located at the centre of the cluster. Three Zn ions in Zn_3_O adopt different coordination geometries: Zn5 resides in a distorted tetrahedral geometry, whereas Zn6 and Zn7 adopt a square pyramidal and an octahedral geometry respectively (Fig. 1a). The tetranuclear cluster consists of two ZnO4 tetrahedra, a ZnO5 square pyramid and a ZnO6 octahedron sharing a central µ_4_-O atom (Fig. 1b). The Zn–O bond lengths and angles varied in the normal ranges of 1.885(9)–2.382(16) Å and 83.0(5)–176.6(5)°, respectively. The average Zn···Zn separation in the Zn_3_O cluster is 3.357 Å which is slightly larger than that in the Zn_4_O cluster (3.196 Å). The BTC^3−^ ligands adopt four different linking modes, denoted as I (linking three Zn_4_O clusters), II (linking two Zn_4_O and a Zn_3_O clusters), III (linking two Zn_3_O and a Zn_4_O cluster) and VI (linking three Zn_3_O clusters) (Fig. 1c) to connect Zn_3_O and Zn_4_O clusters into a large polyhedral cage (Fig. 1d). This cage is composed of nine Zn_3_O and nine Zn_4_O cluster vertexes linked by twenty-four triangular BTC^3−^ ligands and may enclosed a sphere of *ca*. 18.5 Å diameter.

A better insight into this cage can be achieved through connecting the Zn clusters which generates a slightly distorted octahedron whose faces are each composed of four small triangular faces. The size of the octahedral cage is *ca*. 30.7 × 21.5 × 21.5 Å (Fig. 1e). It has been known that the C_3_-symmetric ligand H_3_BTC is useful for the construction of Zn-BTC octahedral cages. However, the short spacer of H_3_BTC usually leads to small cages^32–35^. While increasing the lengths of C_3_-symmetric ligands can afford large cages^36^, this work demonstrates that the Zn-BTC octahedral cage can also be expanded with Zn_3_O and Zn_4_O cluster nodes. In **1**, each octahedral cage serves as a 6-connected octahedral node and shares two edge-fused triangles of six faces with six surrounding octahedra (Fig. 1f), generating a complicated 3D microporous framework (Fig. 1g). Topologically, the Zn_3_O and Zn_4_O clusters can be considered as distorted square pyramidal and octahedral SBUs (5- and 6-connected nodes, respectively), and the BTC^3−^ linker as a triangular unit (3-connected node) (Fig. S2a and b). Thus, the 3D framework of **1** can be viewed as a (3, 5, 6)-connected net (Fig. S2c and d).

To prove the phase purity of the bulk sample, PXRD analysis is performed. The peak positions of the simulated pattern closely match those of the experimental one, indicating phase purity of the as-synthesized sample (Fig. S3). Thermogravimetric analysis (TGA) of **1** shows a mass loss of *ca*. 25.2% from 30 to 400 °C, which is corresponding to the loss of lattice solvent molecules and the coordinated water molecules (calcd. 25.6%). Upon further heating the framework starts to decompose (Fig. S4).

### Tunable luminescence and white light emission

**Figure 2:**
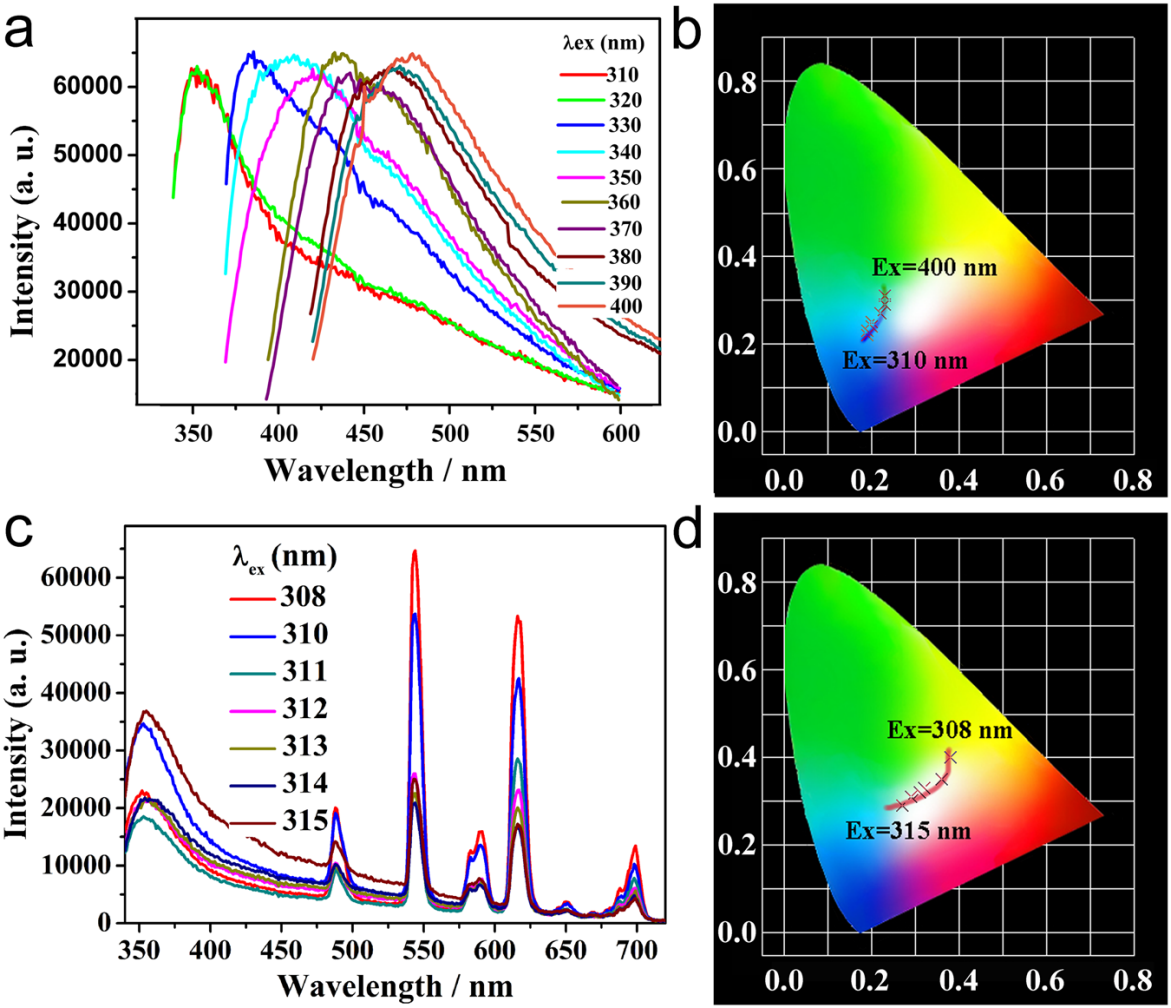
(**a**) The emission spectra of **1** under excitation at various wavelengths; (**b**) The CIE values of **1** at different excitation wavelengths; (**c**) The luminescence emission spectra of Eu^3+^/Tb^3+^-**1** by varying the excitation wavelength; (**d**) The CIE values of Eu^3+^/Tb^3+^-**1** excited at different wavelengths.

Compounds with d^10^ metal centres and organic ligands are desirable candidates for luminescence-emitting materials. Hence luminescence excitation and emission spectra of **1** and H_3_BTC were investigated at room temperature (Fig. S5). Compound **1** and H_3_BTC exhibit emission bands at 422 nm (*λ*_ex_ = 355 nm) and 430 nm (*λ*_ex_ = 340 nm). By comparison with the free ligand, the emission of **1** is blue shifted by *ca*. 8 nm. Such behaviour could be due to the strong electrostatic interaction between the Zn^2+^ ion and BTC^3−^. The solid-state luminescence of **1** excited with various wavelengths was also investigated. As shown in Fig. 2a, as the excitation wavelength varies from 310 to 471 nm, the luminescence colour changes from light-blue to blue-green (Fig. 2b). This result gives us an opportunity to obtain white light emission by incorporating red and green emitting components such as Eu^3+^ and Tb^3+^ into the pore of **1**.

In order to make Ln^3+^-incorporated complexes, compound **1** was immersed in turn in an ethanol solution of Eu^3+^ and an ethanol solution of Tb^3+^, then the solid was filtered and washed by ethanol and diethyl ether several times to remove any residual Eu^3+^ and Tb^3+^ ions on the surface. By adjusting the immersion time, the encapsulated amount of Eu^3+^ and Tb^3+^ can be optimized to achieve white light emission. The resultant Ln^3+^-incorporated complex, namely Eu^3+^/Tb^3+^-**1** contains 0.251% of Eu^3+^ and 0.269% of Tb^3+^, as confirmed by ICP results. The solid-state emission spectrum of Eu^3+^/Tb^3+^-**1** exhibits the characteristic emission peaks of Eu^3+^ (^5^D_0_ to ^7^F_J_, J = 0–4) and Tb^3+^ (^5^D_4_ to ^7^F_J_, J = 6–0) (Fig. 2c). Notably, the CIE coordinates of Eu^3+^/Tb^3+^-**1** excited at 312 nm is (0.32, 0.33), which are very close to those for pure white light (0.333, 0.333), according to the 1931 CIE coordinate diagram. Meanwhile, the emission of Eu^3+^/Tb^3+^-**1** under different excitation wavelengths was also investigated. When excited at 308 nm, the CIE index of Eu^3+^/Tb^3+^-**1** is (0.38, 0.40), and it shows yellow light emission. As the excitation wavelength increases gradually, the main emission peaks of Eu^3+^ and Tb^3+^ gradually weaken. When excited at 315 nm, the CIE of Eu^3+^/Tb^3+^-**1** is (0.27, 0.29), and it displays a blue-green light. As a result, the luminescence colour of Eu^3+^/Tb^3+^-**1** at different excitation wavelengths changes from yellow to white, and eventually becomes blue-green (Fig. 2d).

### Luminescence sensing for metal ions

**Figure 3:**
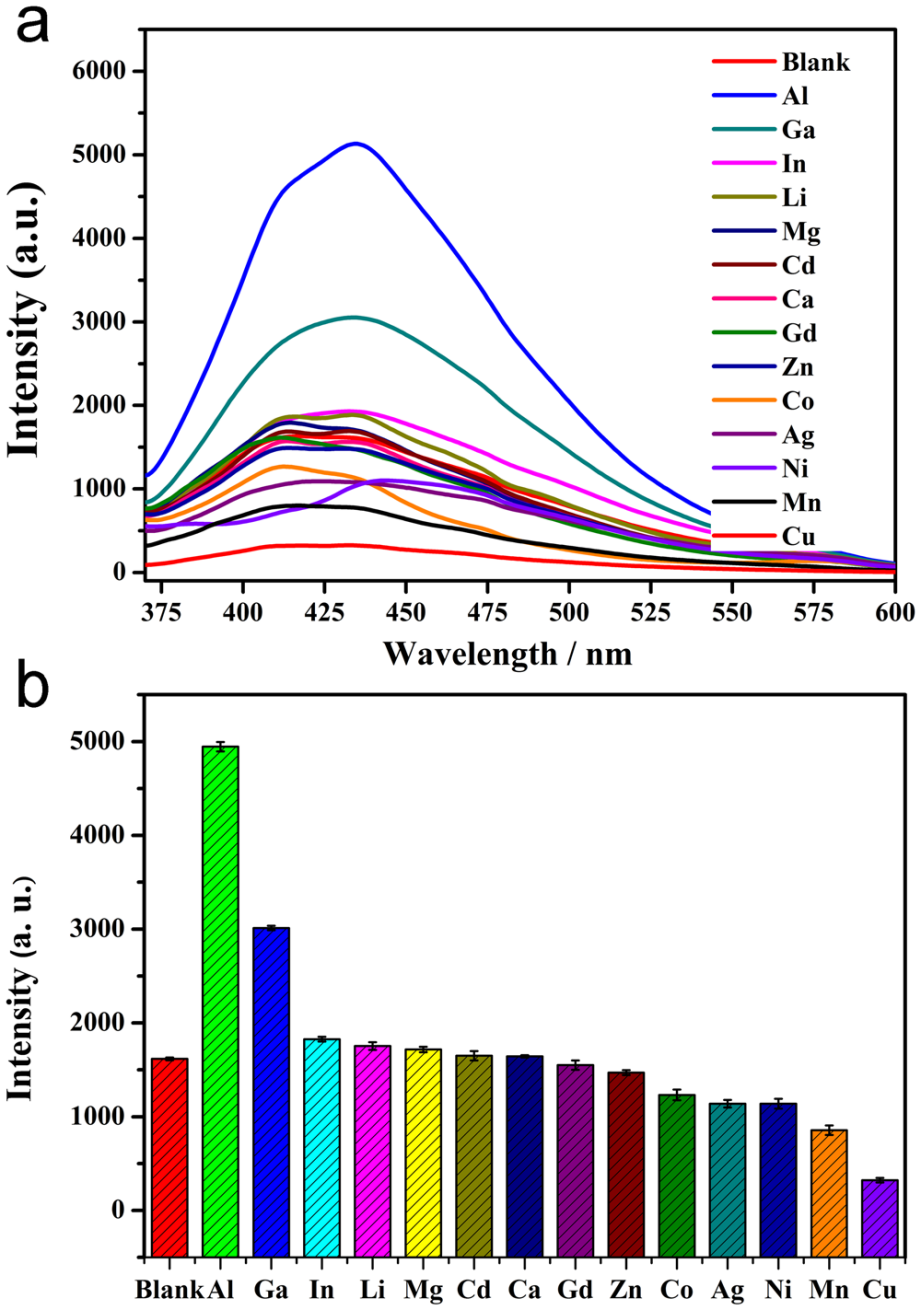
The luminescence spectra (**a**) and intensity (**b**) of **1** after treatment with different metal ions.

The existence of a porous structure makes compound **1** a promising candidate for sensing and detecting metal ions. To investigate the luminescence quenching or enhancement behaviour of **1** by various metal ions, solid samples of **1** were immersed in ethanol solutions containing 0.03 M of M(NO_3_)_n_ (M = Al^3+^, Ga^3+^, In^3+^, Li^+^, Mg^2+^, Cd^2+^, Ca^2+^, Gd^3+^, Zn^2+^, Co^2+^, Ag^+^, Ni^2+^, Mn^2+^, Cu^2+^, n = 1−3) for one hour and then ultrasonically agitated for 20 min to form a metal-ion-incorporated MOF suspension. The corresponding luminescence spectra are recorded and are compared in Fig. 3a. The emission spectra show that the luminescence intensity of M^n+^-**1** excited at 355 nm varies significantly depending on the identity of the metal ions. For example, Li^+^, Mg^2+^, Ca^2+^, In^3+^, Zn^2+^, Cd^2+^ and Gd^3+^ have only a slight effect on the luminescence intensity after incorporation into the pores, whereas the other metal ions have varying degrees of effects. Among them the Cu^2+^ ion has a significant quenching effect on the emission of **1**. The descending order of the quenching efficiencies of the metal ions is as follows: Cu^2+^ > Mn^2+^ > Ni^2+^ > Ag^+^ > Co^2+^ > In^3+^ > Li^+^ > Mg^2+^ > Zn^2+^ > Cd^2+^ > Ca^2+^ > Gd^3+^. In contrast to Cu^2+^, the Al^3+^ and Ga^3+^ ions show significant enhancement on the emission intensity. Particularly in the presence of Al^3+^, the emission intensity is about three times than the metal-ion free **1** (Fig. 3b). These results clearly indicate that **1** shows a high selectivity towards Cu^2+^ and Al^3+^.

**Figure 4:**
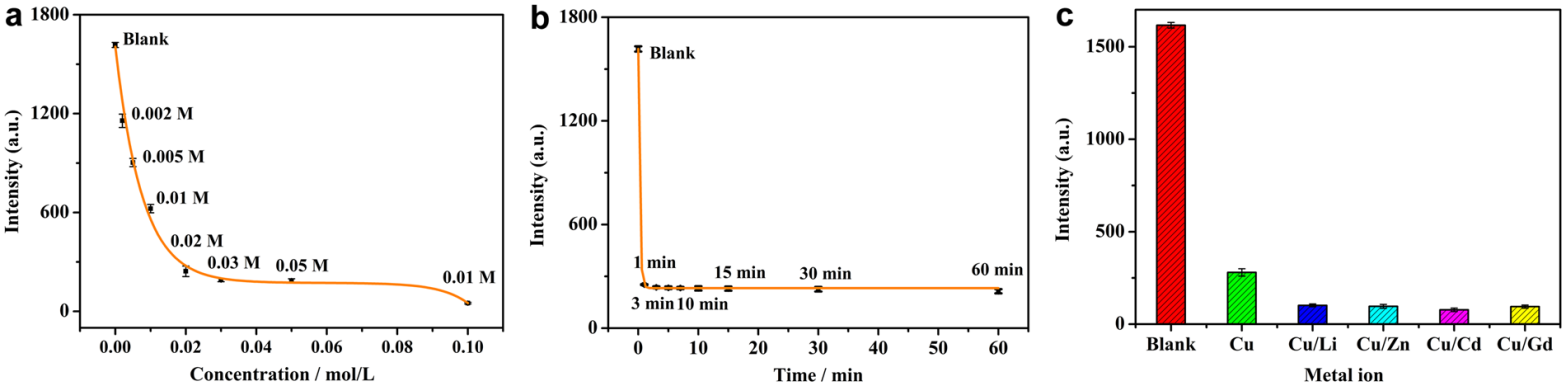
The luminescence intensity of **1** (**a**) after immersion in ethanol solutions of different concentrations of Cu^2+^ ions (**b**) after immersion in a 0.03 M ethanol solution of Cu^2+^ for various time periods and (**c**) after immersion in ethanol solutions with different metal ions.

The relationship between the luminescence intensity and the concentration of Cu^2+^ has been investigated by measuring the emission spectra of **1** after immersion in solutions of various concentrations of Cu^2+^ ions (Fig. S6a). The results show that the luminescence intensity of Cu^2+^-incorporated complex is greatly dependent on the concentration of the metal ion. The luminescence intensity decreases quickly as the concentration of Cu^2+^ increases and it remains unchanged when the Cu^2+^ concentration is greater than 0.03 M (Fig. 4a). Unlike Cu^2+^ ion concentration, the immersion time seems to have no influence on the luminescence intensity. As shown in Fig. 4b, the luminescence intensity of **1** after being immersed in 0.03 M Cu^2+^ ethanol solution for less one minute decreases sharply and it is also observed that prolongation of immersion time up to 60 min does not cause any further decrease of the luminescence intensity (Fig. S6b). Furthermore, this selective detection of Cu^2+^ is not influenced by the existence of other metal ions such as Li^+^, Zn^2+^, Cd^2+^ and Gd^3+^ (Figs 4c and S6c). A good linear correlation between (I_0_−I)/I and the concentration of Cu^2+^ is observed with the *K*_sv_ value of 286.1 M^−1^ (Fig. S6d). The detection limit is calculated on the basis of 3*σ*/*k* to be 1.34 × 10^−3^ M.

The possible mechanism of luminescence quenching by Cu^2+^ could involve the binding of Cu^2+^ through Lewis acid-base interaction, as suggested for the selective sensing of Cu^2+^ ion with microporous frameworks, such as [Cd_2_(PAM)_2_(dpe)_2_(H_2_O)_2_]·0.5(dpe)^37^ and {Mg(DHT)(DMF)_2_}^38^ (H_2_PAM = 4,4-methylenebis(3-hydroxy-2-naphthalene-carboxylic acid), dpe = 1,2-di(4-pyridyl)ethylene, DHT = 2,5-dihydroxyterephthalate). Such binding reduces the intraligand luminescent efficiency and results in the quenching effect^37–40^. In the case of Al^3+^ sensing, decomposition of **1** occurred due to hydrolysis of Al^3+^, which made the solution acidic. In 0.03 M Al^3+^ solution, **1** was partially dissolved, which released BTC^3−^ in solution and thereby enhancing the ligand fluorescence^41^.

The luminescence quenching of Cu^2+^ may also result from partially exchange of the metal ions from Zn^2+^ to Cu^2+^ in the framework. To check the results, **1** was immersed in ethanol containing 0.03 M Cu^2+^ for varying periods of time. The filtered powder was washed thoroughly with ethanol until the filtration became colourless. The colour of crystals changes from colourless to green after exchange with Cu^2+^. The Cu^2+^-exchanged samples thus obtained were then subjected to ICP analysis. The results showed that with immersion time increasing from 1 min to 24 h, the Cu-exchange level on the framework gradually increased accompanied by a reduction of Zn content in the compound. As indicated in Table S1, approximately 50% of framework was replaced with Cu^2+^ ion within one day and nearly complete replacement of Cu^2+^ (96%) took place after two weeks. Surprisingly, the reversed ion exchange failed and so did the exchange with other transition metal ions like Co^2+^ and Ni^2+^. SEM images (Fig. S7) reveal that after Cu^2+^ exchange, the large crystals of **1** (ca. 300 μm) with a regular shape collapse into microcrystalline solid. However, the XRD pattern of the Cu^2+^-exchanged samples shows similar peaks to those of **1**, suggesting that the framework structure remains intact after MOF exchange with Cu^2+^ ion (Fig. S3). Moreover, XPS measurements were also carried out to confirm the existence of Cu in the Cu^2+^-exchanged sample. Fig. S8 shows that the Cu 2p 3/2 and 1/2 spectra of Cu^2+^-exchanged sample were located at 934 and 954 eV, respectively, both of which suggest the presence of Cu^2+^ in the Cu^2+^-exchanged sample.

### Gas adsorption properties of the Cu^2+^-exchanged compound

**Figure 5:**
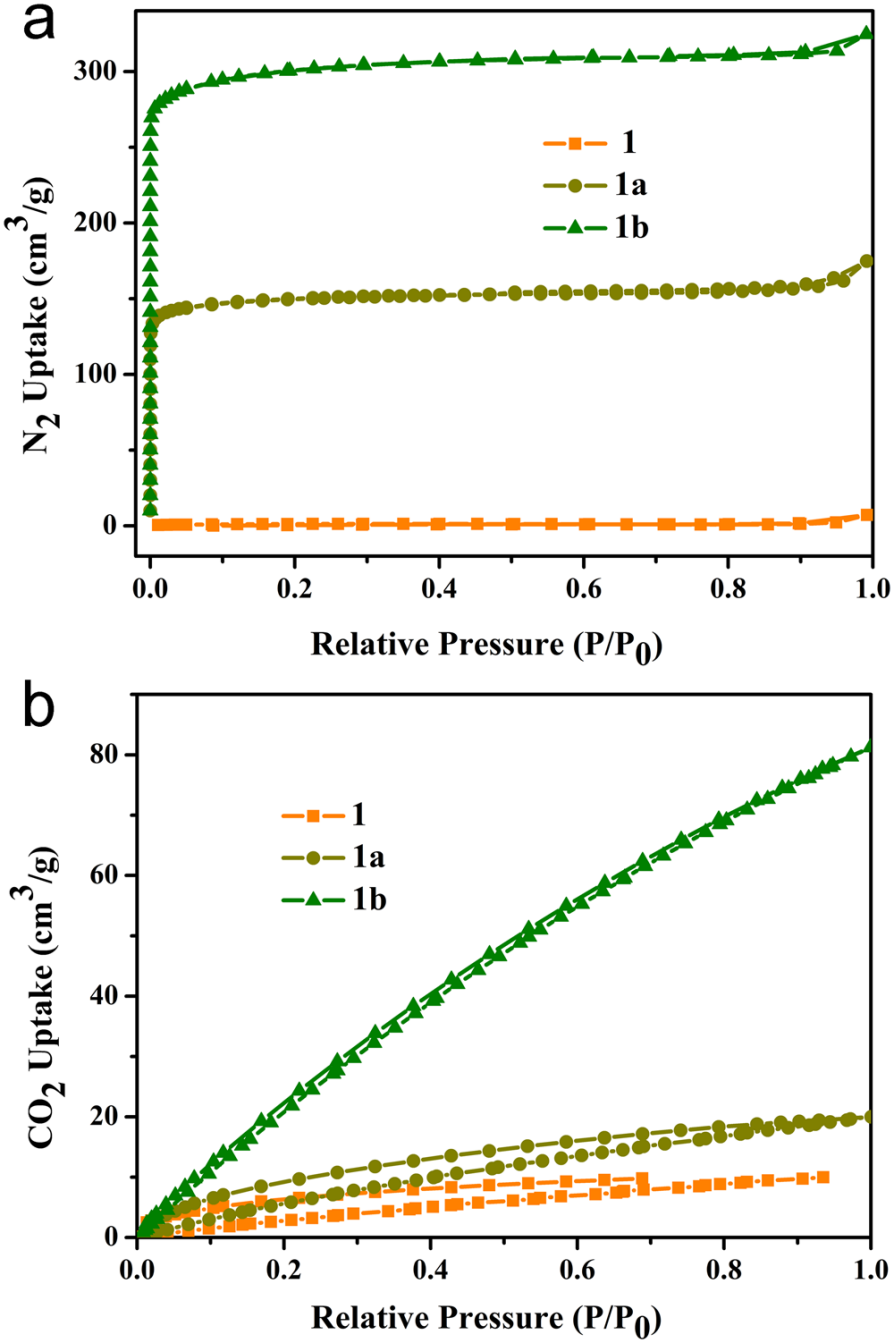
(**a**) N_2_ adsorption-desorption isotherms of **1**, **1a** and **1b** at 77 K; (**b**) CO_2_ adsorption-desorption isotherms of **1**, **1a** and **1b** at 273 K.

Adsorption experiments were carried out to investigate the porosity. The samples were degassed at 100 °C for 12 h under vacuum prior to gas adsorption/desorption measurements. The activated sample **1** shows no significant adsorption for N_2_ and CO_2_, presumably due to the pore collapse during sample activation. Interestingly, the adsorption capacity for either N_2_ or CO_2_ substantially increases by replacing Zn^2+^ with Cu^2+^ in the framework. The N_2_ adsorption of the Cu^2+^-exchanged samples with 50% (**1a**) and 96% (**1b**) exchange ratios at 77 K exhibits a type I isotherm, typical for materials that show permanent microporosity. The highest adsorbed amount of N_2_ is 174.8 cm^3^ g^−1^ for **1a** and 324.6 cm^3^ g^−1^ for **1b**, and the corresponding pore volumes are 0.245 and 0.485 cm^3^ g^−1^ respectively (Fig. 5a). The Langmuir and Brunauer-Emmett-Teller (BET) surface areas are 649.02 and 589.05 m^2^g^−1^ for **1a** and 1299.33 and 1179.73 m^2^g^−1^ for **1b**. The CO_2_ adsorption capacity increases from 38.5 cm^3^ g^−1^ for **1a** to 136.1 cm^3 ^g^−1^ for **1b**. In both case, the amount of CO_2_ uptake decreases by 40.3% as the temperature increases from 273 to 298 K, indicating a typical physisorption behavior (Figs 5b and S9). These results demonstrate that the increase of Cu-exchange ratio dramatically enhance the adsorption capacity for N_2_ and CO_2_. The main reason for this may be due to the fact that the replacement of Zn^2+^ by Cu^2+^ enhances the framework robustness thereby improving the adsorption properties^42^.

## Conclusion

In summary, a new cage-based MOF with two different types of Zn cluster SBUs has been synthesized and structurally characterized. This compound features a large octahedral cage constituted by nine Zn_3_O and nine Zn_4_O clusters and twenty-four triangular BTC^3−^ ligands. Tunable luminescence and white light emission can be achieved by changing the excitation wavelength and by incorporation of Eu^3+^/Tb^3+^ ions into the compound. While other transition metal ions such as Mn^2+^, Co^2+^ and Ni^2+^ displayed relatively weak quenching effects, only Cu^2+^ and Al^3+^ ions showed significant changes in the emission spectra, which demonstrates that **1** could be regarded as a potential material for selective sensing of Cu^2+^ and Al^3+^ ions. In addition, the facile ion exchange with Cu^2+^ without loss of structural integrity as described herein provide an post-synthesis route to construct isomorphous Cu-MOF that cannot be obtained by direct synthesis.

## Methods

### Materials and instrumentation

All chemicals were purchased commercially and used as received. TGA was performed using a TGA/NETZSCH STA449C instrument heated from 30–800 °C (heating rate of 10 °C/min, nitrogen stream). IR spectrum using a KBr pellet was recorded on a Spectrum-One FT-IR spectrophotometer in the range 4000-400 cm^−1^. The powder X-ray diffraction (PXRD) patterns were recorded on crushed single crystals in the 2θ range 5–55° using Cu K*α* radiation. ICP elemental analyses for the metal ions were performed with an ultima2 X-ray ICP optical emission spectrometer. Elemental analyses for C and H were measured with Elemental Vairo EL III Analyser. Luminescence spectra for the solid samples were recorded on an Edinburgh Analytical instrument FLS920. Luminescence spectra for the liquid samples were recorded on a HITACHI F-7000. Gas adsorption measurements were performed in an ASAP (Accelerated Surface Area and Porosimetry) 2020 System. SEM images were obtained using a Phenom G2 SEM microscope.

### Preparation of compound 1

A mixture of Zn(NO_3_)_2_·6H_2_O (148.7 mg, 0.5 mmol), H_3_BTC (103.5 mg, 0.5 mmol) and 4-cyanopyridine (104.1 mg, 1.0 mmol) in ethanol (10 mL) was heated in a Teflon-lined stainless steel vessel (24 mL) at 110 °C for three days and then cooled to room temperature in two days. The resulting colourless crystals of **1** were obtained and washed several times with ethanol (yield 56% based on Zn). Elemental analysis calcd. (%) for **1** C_47_H_66_O_37_Zn_7_ (1680.58): C 33.56, H 3.93; found: C 33.29, H 3.87. IR (cm^−1^) (Fig. S9): 3433 s, 2977 vs, 1687 w, 1574 s, 1440 s, 1365 vw, 1260 w, 1197 vw, 1105 w, 1046 w, 926 w, 875 w, 829 w, 762 s, 730 s, 553 w, 469 w.

### Preparation of Eu^3+^/Tb^3+^-1

The Ln^3+^-incorporated complex was prepared by first soaking a sample of **1** (35 mg) in an ethanol solution (3 mL) containing Tb(NO_3_)_3_·6H_2_O (20 mg) for two hours, afterwards in a Eu(NO_3_)_3_·6H_2_O (20 mg) ethanol solution (3 mL) for another two hours. Then the crystals were collected, washed thoroughly with ethanol and diethyl ether, and dried in air to afford Eu^3+^/Tb^3+^-1.

### Immersion experiments of 1 with different metal ions

Compound **1** (30 mg) was immersed in 0.03 M solutions of M(NO_3_)_n_ in ethanol at room temperature for one hour (M = Al^3+^, Ga^3+^, In^3+^, Li^+^, Mg^2+^, Cd^2+^, Ca^2+^, Gd^3+^, Zn^2+^, Co^2+^, Ag^+^, Ni^2+^, Mn^2+^, Cu^2+^, n = 1−3) and then ultrasonically agitated for 20 min to form a metal-ion-incorporated MOF suspension.

### Single-crystal structure determination

Single-crystal X-ray diffraction data were collected on a Rigaku Diffractometer with a Mercury CCD area detector (Mo K*α*; *λ* = 0.71073 Å) at room temperature. Empirical absorption corrections were applied to the data using the Crystal Clear program^43^. The structure was solved by direct methods using SHELXS-97^44^ and refined by full-matrix least-squares on *F*^2^ using SHELXL-2016 program^45^. Metal atoms were located from the *E*-maps, and other non-hydrogen atoms were located in successive difference Fourier syntheses. All non-hydrogen atoms were refined anisotropically. The organic hydrogen atoms were positioned geometrically. Since the position of the disorder water molecules could not be resolved from Fourier maps, PLATON/SQUEEZE^46^ was used to compensate the data for their contribution to the diffraction patterns. The SQUEEZE calculations showed a total solvent accessible area volume of 10178 Å^3^ in **1** and the residual electron density amounted to 1744 e per unit cell, corresponding to about seven ethanol molecules per asymmetric unit. The final formula was then calculated from the TGA result combined with elemental analysis data. Crystallographic data and other pertinent information for **1** are summarized in Table S2. Selected bond distances and angles are listed in Table S3. CCDC number for **1** is 1542054.

## Electronic supplementary material

Supplementary Information
