# Supplementary material for: A Multifunctional Nanocage-based MOF with Tri- and Tetranuclear Zinc Cluster Secondary Building Units
Source: Sci Rep. 2018 Feb 15;8:3117. doi: 10.1038/s41598-018-21382-1 (PMC5814399; doi:10.1038/s41598-018-21382-1)
Supplement: Supplementary file 1 — Supplementary Information [file 41598_2018_21382_MOESM1_ESM.doc]

**Supplementary Information for**

**A Multifunctional Nanocage-based MOF with Tri- and Tetranuclear Zinc Cluster Secondary Building Units**

Zhongyuan Zhou^1^, Xiushuang Xing^1,2^, Chongbin Tian^1^, Wei Wei^1,2^, Dejing Li^1,2^, Falu Hu^1,2^, and Shaowu Du^1,*^

^1^State Key Laboratory of Structural Chemistry, Fujian Institute of Research on the Structure of Matter, Chinese Academy of Sciences, Fuzhou, 350002, P. R. China.

^2^University of Chinese Academy of Sciences, Beijing, 100039, P. R. China.

[*swdu@fjirsm.ac.cn](mailto:*swdu@fjirsm.ac.cn)


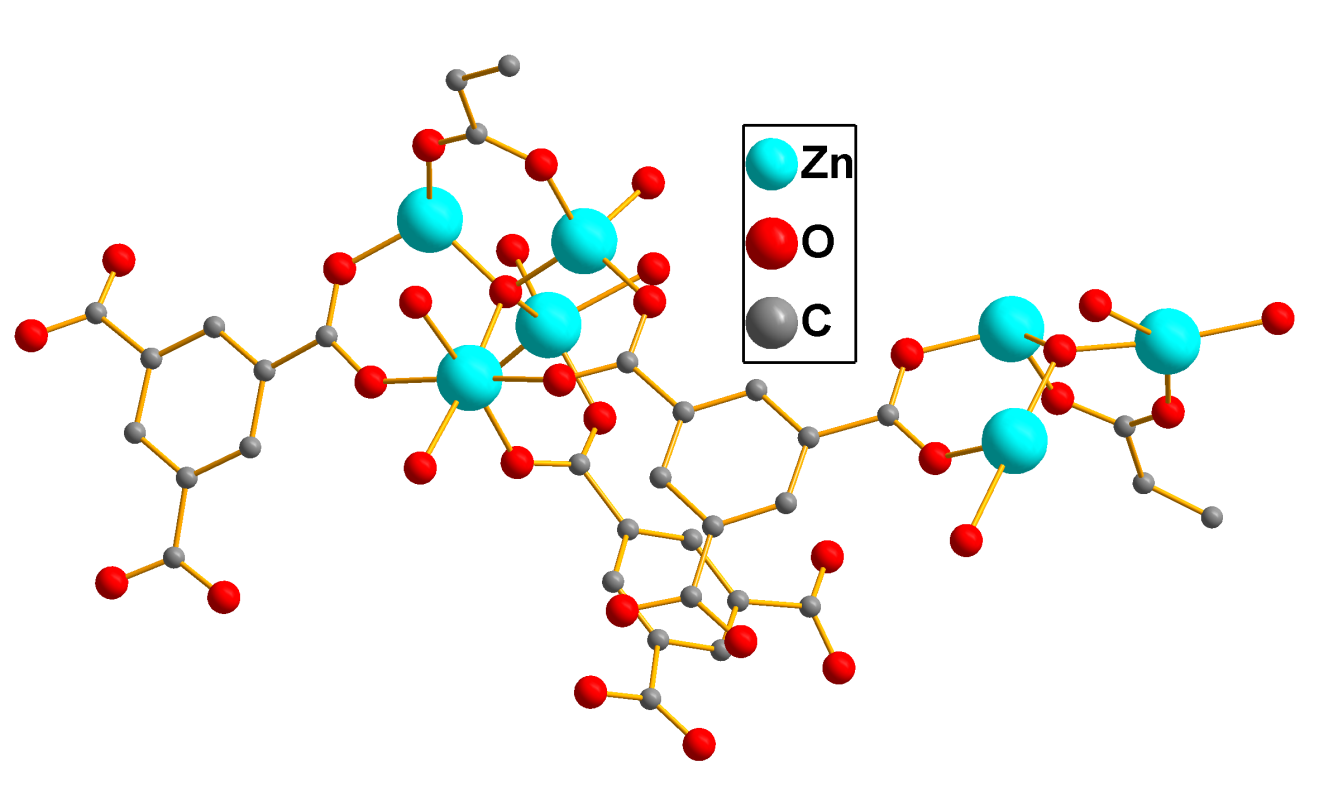


**Figure S1.** The asymmetric unit of compound **1**.


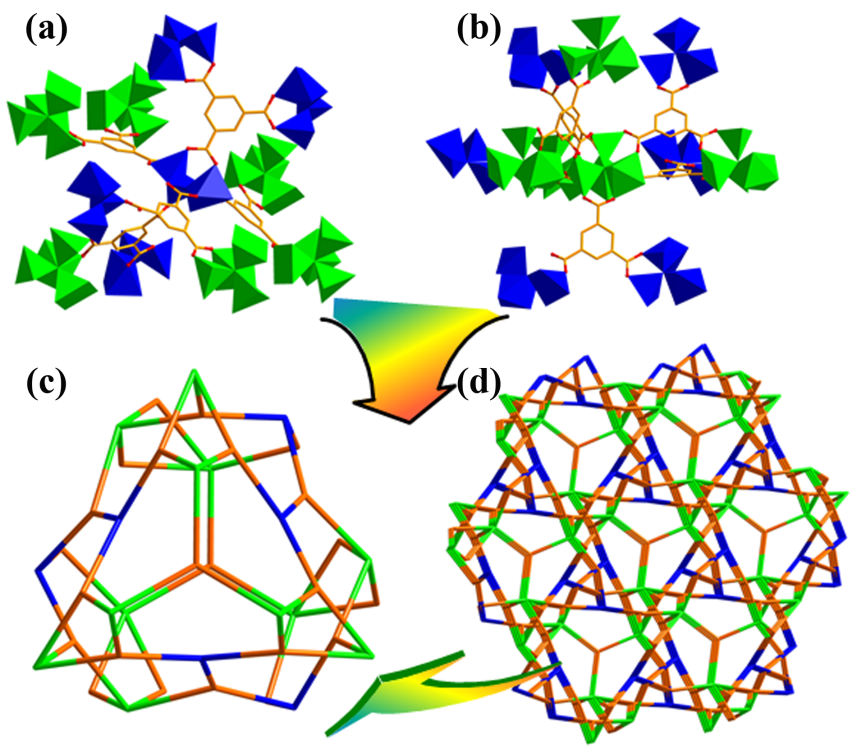


**Figure S2.** (a), (b) Schematic representation for the Zn_3_O and Zn_4_O units. (c), (d) (3, 5, 6)-connected topological net of **1**.


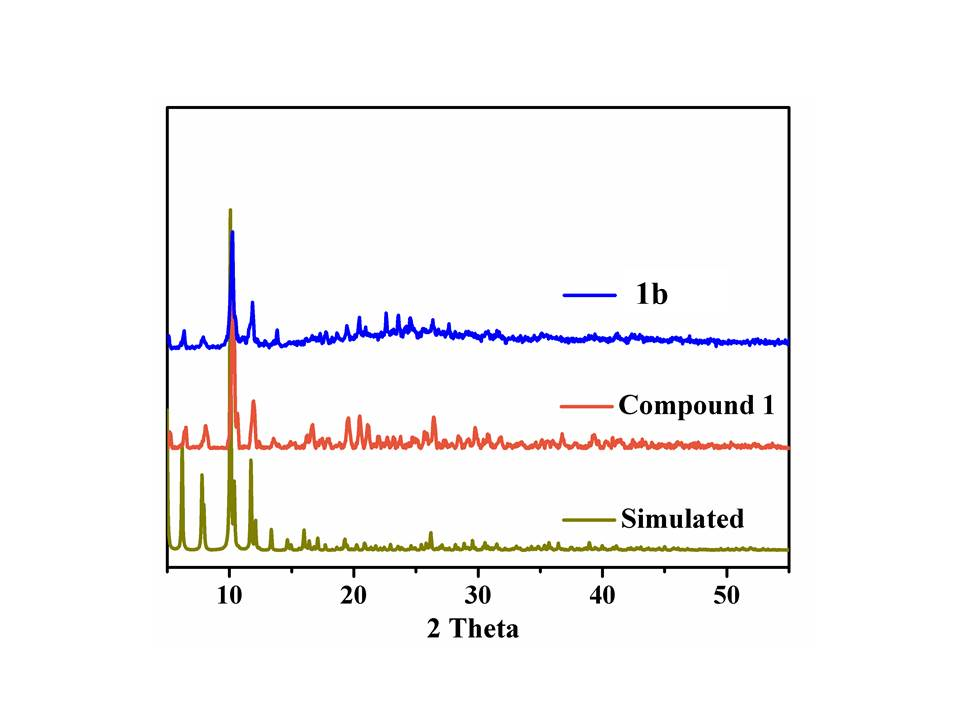


**Figure S3.** Simulated and experimented X-ray powder patterns of **1** and Cu^2+^-exchanged compound **1b**.


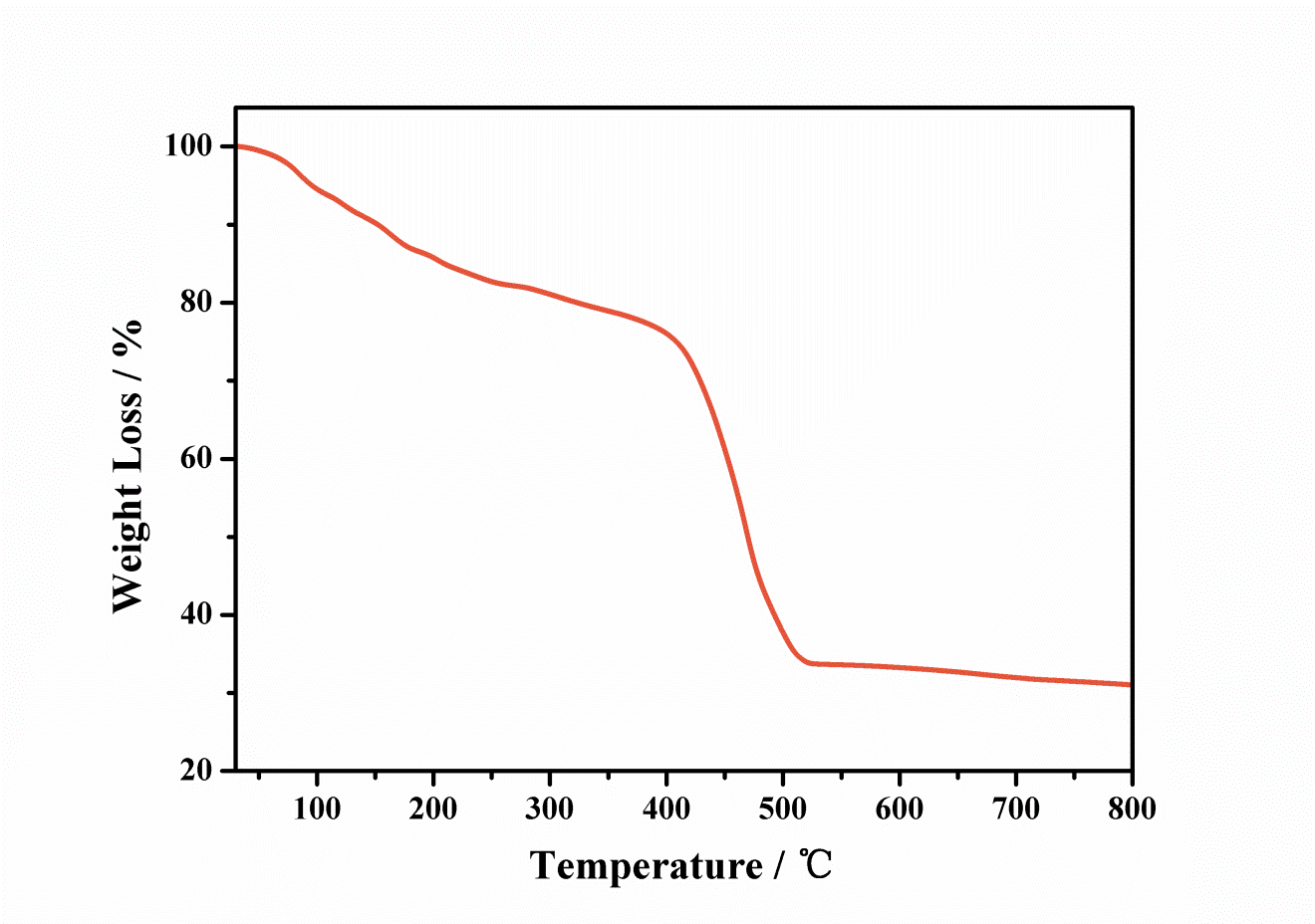


**Figure S4.** TGA curve of compound **1**.


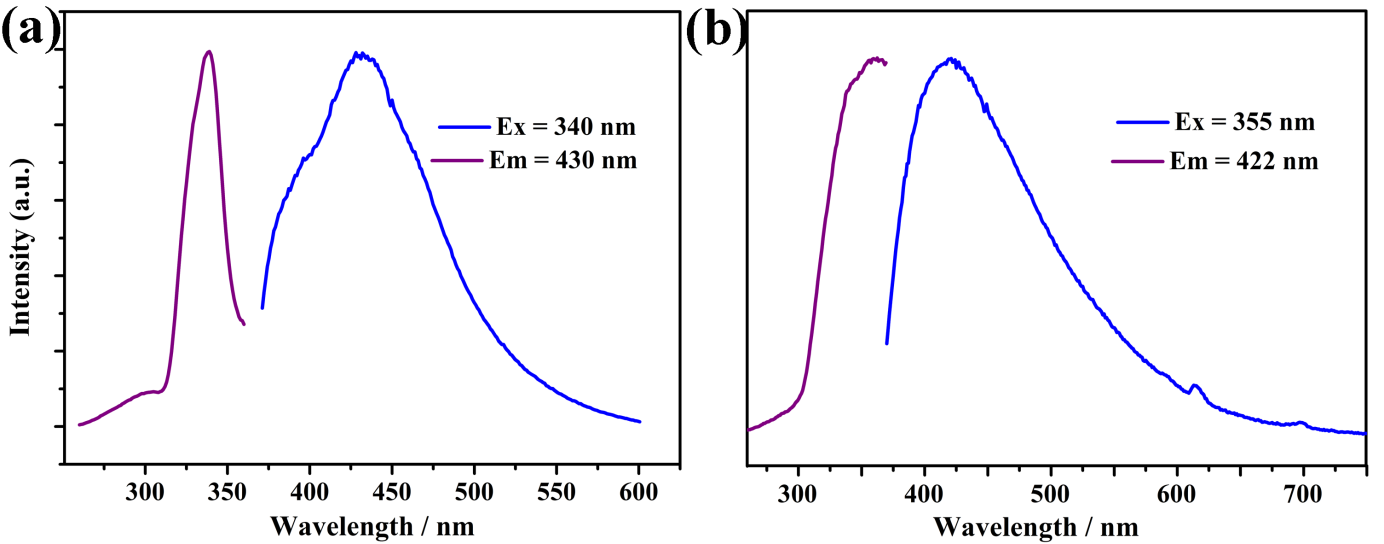


**Figure S5.** Fluorescent excitation and emission spectra of the ligand (H_3_BTC) (a) and **1** (b).


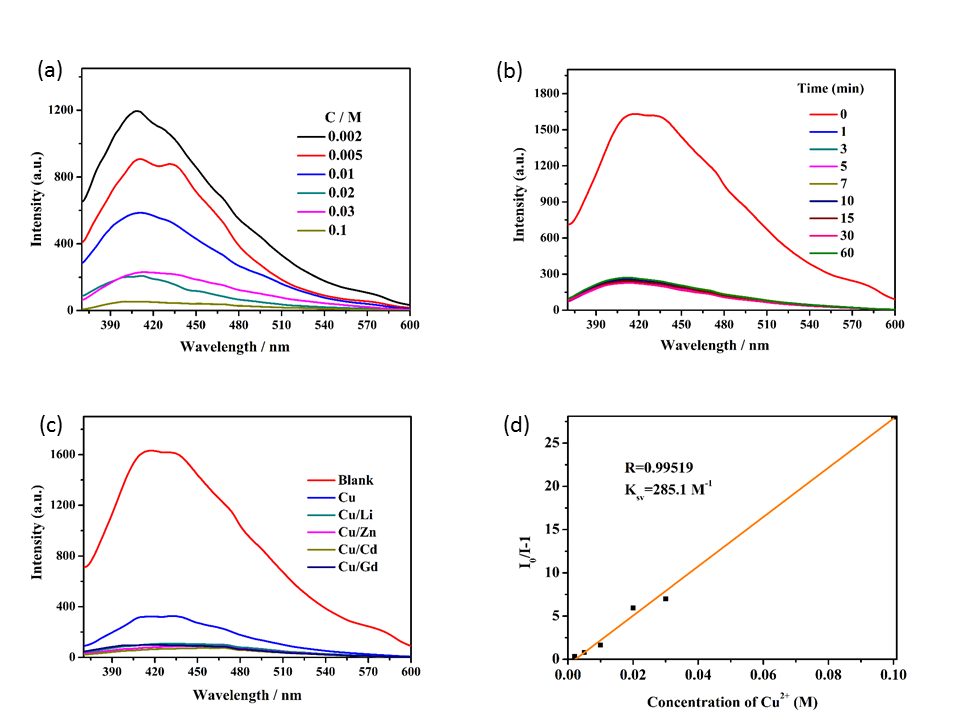


**Figure S6.** The luminescence spectra of **1** (a) after immersion in solutions of different concentrations of Cu^2+^ ions; (b) after immersion in a 0.03 M solution of Cu^2+^ for various time periods; (c) after immersion in ethanol solutions with different metal ions; (d) SV plot of **1** in ethanol solutions with different concentrations of Cu^2+^ ions.


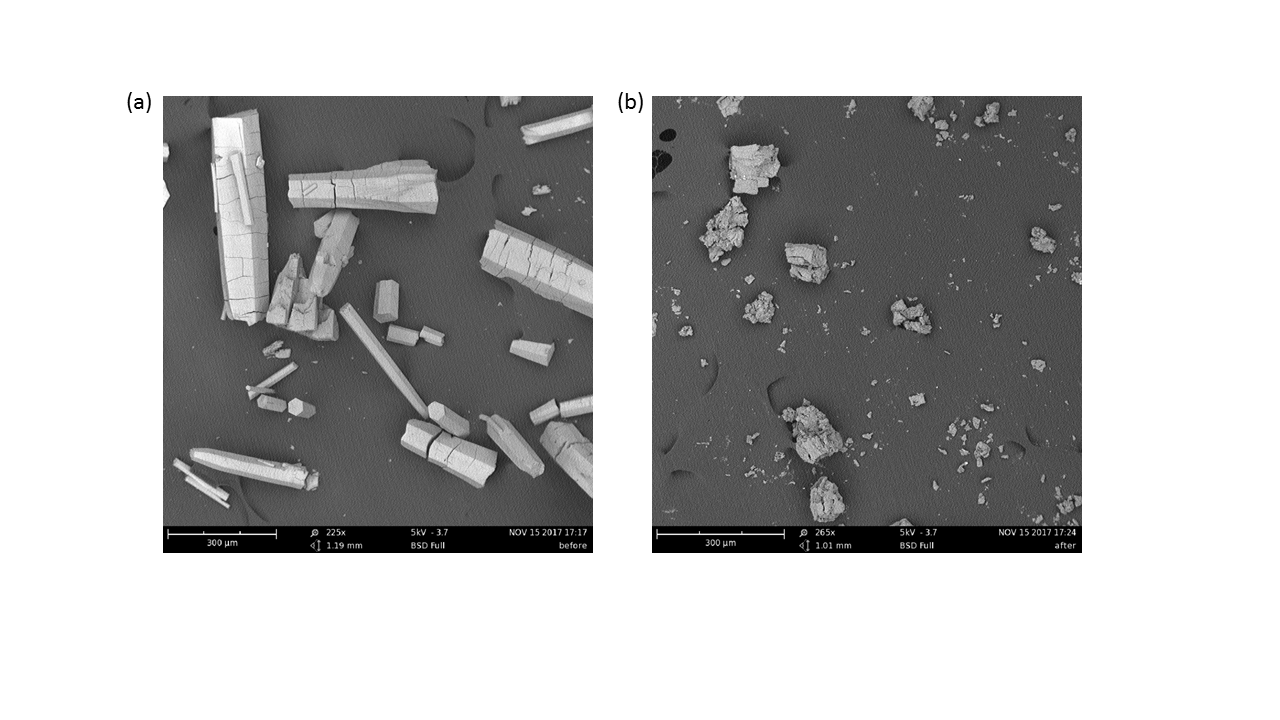


**Figure S7.** SEM images of **1** (a) before Cu^2+^ exchange and (b) after Cu^2+^ exchange for two weeks.


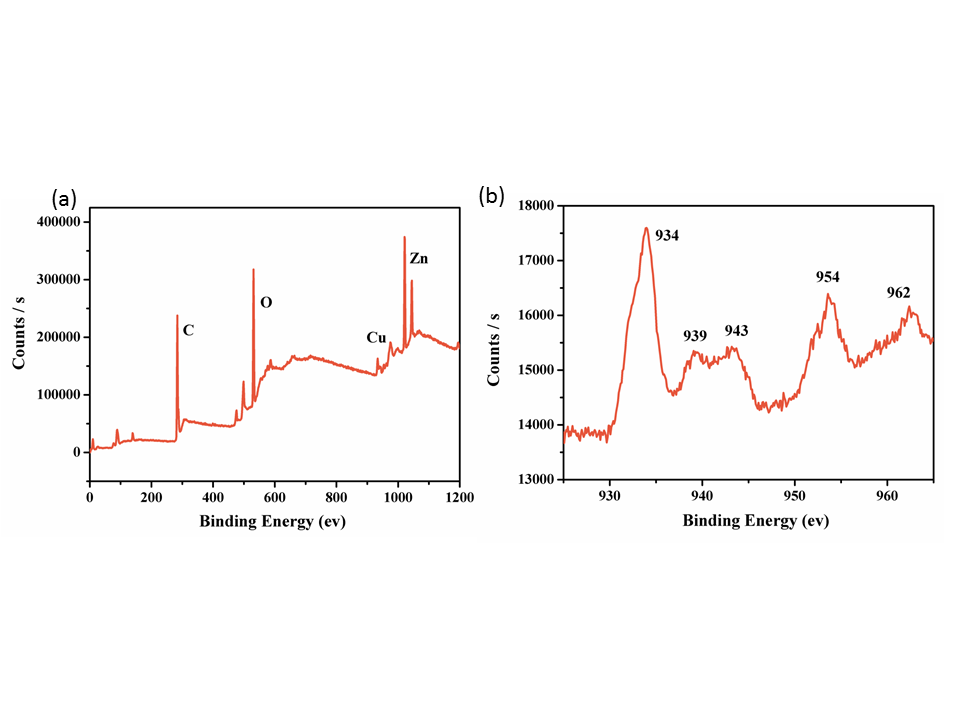


**Figure S8.** (a) XPS of Cu^2+^-exchanged compound; (b) XPS of Cu in the Cu^2+^-exchanged compound.


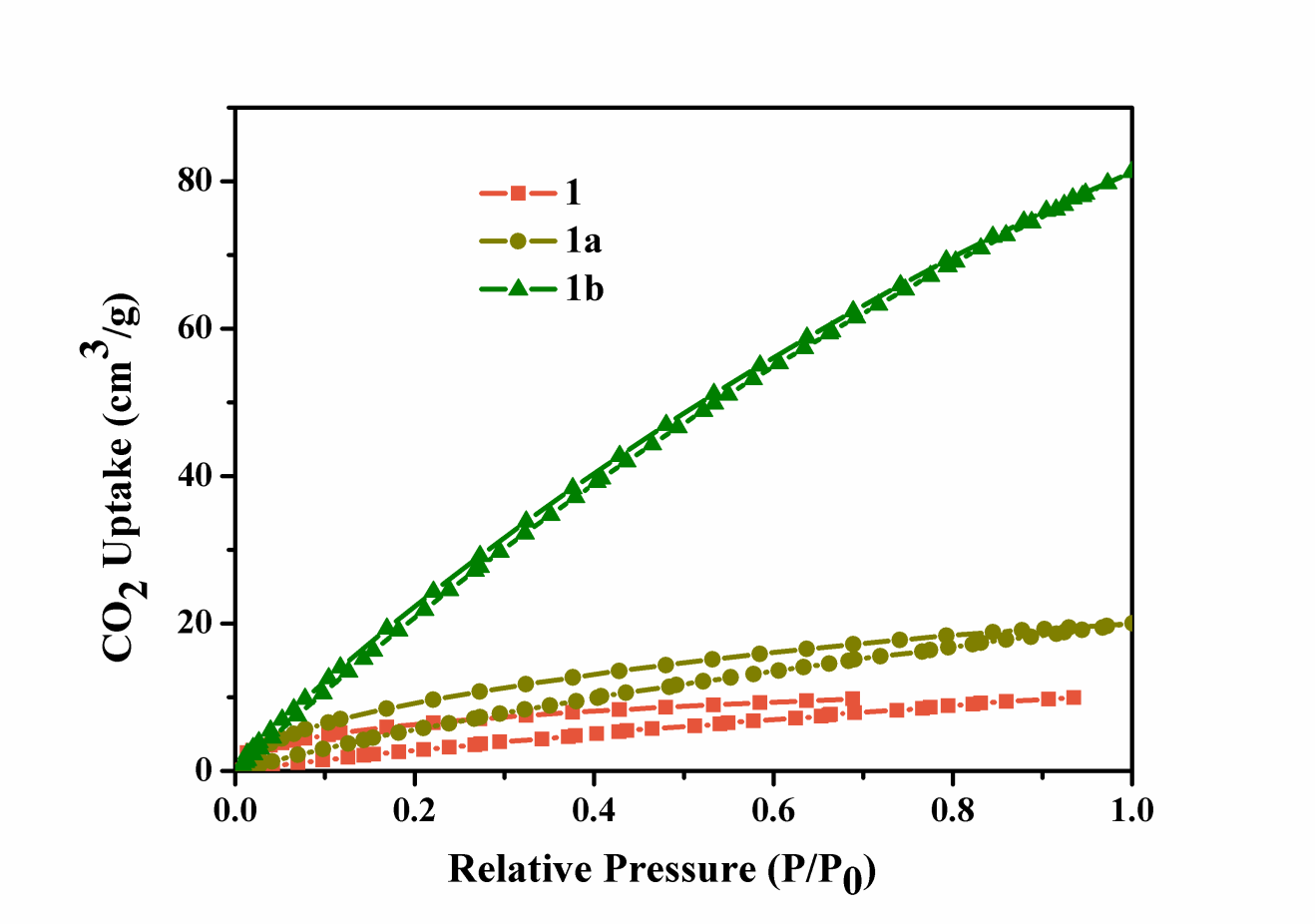


**Figure S9.** CO_2_ adsorption-desorption isotherms of **1**, **1a** and **1b** at 298 K.


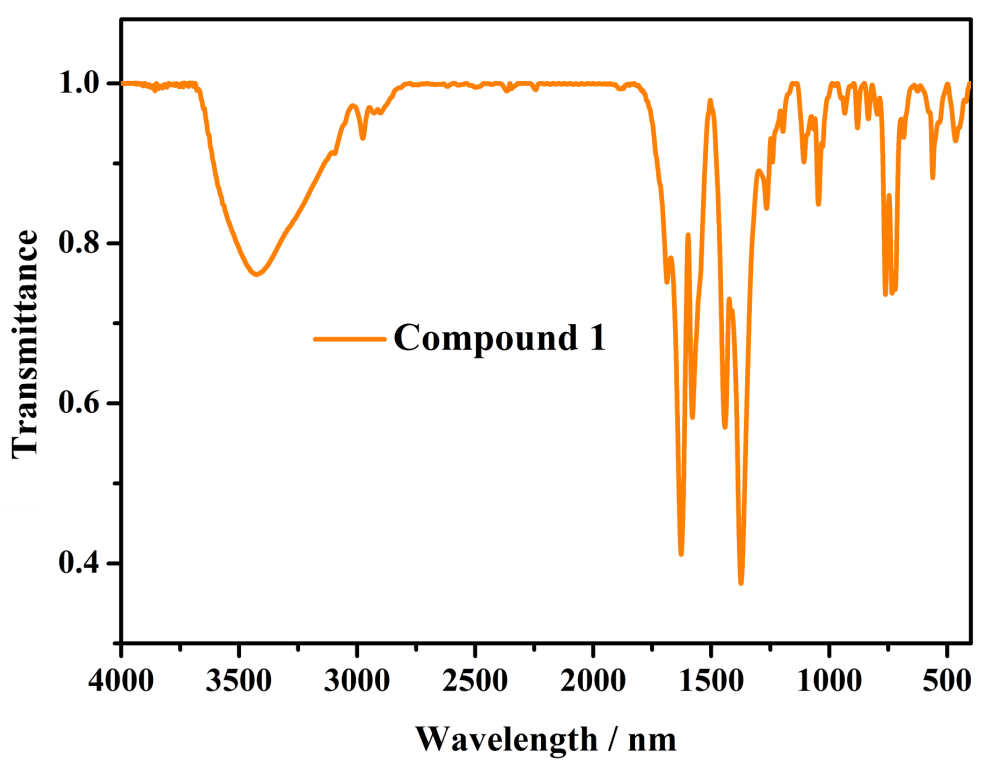


**Figure S10.** IR spectrum of compound **1**.

**Table S1.** Observed Cu^2+^ and Zn^2+^ amount in **1** after being immersed in the ethanol solution containing 0.03 M of Cu^2+^ for various time periods.

| Concentration of Cu^2+^ | The immersed time | Observed Cu^2+^ amount in the sample / % | Observed Zn^2+^ amount in the sample / % |
| --- | --- | --- | --- |
| 0.03 M | 1 min | 0.71 | 22.03 |
|  | 10 min | 1.58 | 19.68 |
|  | 1 h | 2.8 | 18.88 |
|  | 6 h | 5.19 | 17.25 |
|  | 24 h  2 week | 8.41  18.66 | 13.61  0.996 |

**Table S2.** Crystal data and structure refinement of compound **1**.

| Compound | **1** |
| --- | --- |
| CCDC | 1542054 |
| Formula | C_47_H_66_O_37_Zn_7_ |
| Formula weight | 1680.58 |
| Crystal system | Trigonal |
| Space group | *R3* |
| *α*(º) | 90 |
| *β*(º) | 90 |
| *γ*(º) | 120 |
| *a*(Å) | 28.5408(11) |
| *b*(Å) | 28.5408(11) |
| *c*(Å) | 25.5419(18) |
| *V*(Å^3^) | 18018.4(19) |
| *Z* | 9 |
| *D*_calcd_ (g cm^–3^) | 1.394 |
| *μ* (mm^–1^) | 2.138 |
| *F*(000) | 7686 |
| Temperature (K) | 293 |
| Refln. measured | 51641 |
| Independent refln. | 13628 |
| Observed refln. | 9606 |
| *R*_1_^a^ [*I* > 2*σ*(*I*)] | 0.0562 |
| *wR*_2_^b^ | 0.1178 |
| GOF | 1.018 |
| Δ*ρ*_max_/ Δ*ρ*_min_ (e.Å^3^) | 1.00/-0.477 |

^a^ *R*_1_ = ∑ ||*F*_o_| – |*F*_c_||/∑ |*F*_o_|, ^b^ *wR*_2_ = [∑*w*(*F*_o_^2^ – *F*_c_^2^)2/∑*w*[(*F*_o_^2^) ^2^]^1/2^

**Table S3.** Bond length (Å) and Bond angle (º) of compound **1**.

| Zn(1)−O(12)#1 | 1.941(7) | Zn(4)−O(25) | 2.224(12) |
| --- | --- | --- | --- |
| Zn(1)−O(16) | 1.919(7) | Zn(4)−O(26) | 2.168(12) |
| Zn(1)−O(21) | 1.948(7) | Zn(5)−O(6)#2 | 1.946(8) |
| Zn(1)−O(24) | 1.939(6) | Zn(5)−O(13) | 1.947(7) |
| Zn(2)−O(3) | 1.938(6) | Zn(5)−O(19) | 1.948(7) |
| Zn(2)−O(18)#1 | 1.962(6) | Zn(5)−O(23) | 1.918(8) |
| Zn(2)−O(22) | 1.982(7) | Zn(6)−O(5)#2 | 2.019(10) |
| Zn(2)−O(24) | 1.918(6) | Zn(6)−O(7)#3 | 2.006(9) |
| Zn(3)−O(9) | 1.953(7) | Zn(6)−O(20) | 2.003(8) |
| Zn(3)−O(11)#1 | 2.151(9) | Zn(6)−O(23) | 1.976(7) |
| Zn(3)−O(17)#1 | 2.030(7) | Zn(6)−O(27) | 2.038(11) |
| Zn(3)−O(24) | 1.988(6) | Zn(6)−O(28) | 2.323(9) |
| Zn(3)−O(29) | 2.074(16) | Zn(7)−O(2)#4 | 2.015(9) |
| Zn(4)−O(4) | 2.118(10) | Zn(7)−O(8)#3 | 1.885(9) |
| Zn(4)−O(10) | 2.010(9) | Zn(7)−O(14) | 1.962(8) |
| Zn(4)−O(15) | 2.155(8) | Zn(7)−O(23) | 2.102(8) |
| Zn(4)−O(24) | 1.987(6) | Zn(7)−O(30) | 2.382(16) |
|  |  |  |  |
| O(12)#1−Zn(1)−O(21) | 101.5(3) | O(24)−Zn(4)−O(25) | 174.6(4) |
| O(16)−Zn(1)−O(12)#1 | 105.4(3) | O(24)−Zn(4)−O(26) | 89.5(4) |
| O(16)−Zn(1)−O(21) | 109.2(3) | O(26)−Zn(4)−O(25) | 86.0(5) |
| O(16)−Zn(1)−O(24) | 116.9(3) | O(6)#2−Zn(5)−O(13) | 118.7(3) |
| O(24)−Zn(1)−O(12)#1 | 113.5(3) | O(6)#2−Zn(5)−O(19) | 115.8(4) |
| O(24)−Zn(1)−O(21) | 109.2(3) | O(13)−Zn(5)−O(19) | 110.1(4) |
| O(3)−Zn(2)−O(18)#1 | 107.2(3) | O(23)−Zn(5)−O(6)#2 | 100.7(4) |
| O(3)−Zn(2)−O(22) | 121.1(3) | O(23)−Zn(5)−O(13) | 106.3(3) |
| O(18)#1−Zn(2)−O(22) | 101.4(3) | O(23)−Zn(5)−O(19) | 102.9(4) |
| O(24)−Zn(2)−O(3) | 109.9(3) | O(5)#2−Zn(6)−O(27) | 87.1(4) |
| O(24)−Zn(2)−O(18)#1 | 106.7(3) | O(5)#2−Zn(6)−O(28) | 88.1(5) |
| O(24)−Zn(2)−O(22) | 109.3(3) | O(7)#3−Zn(6)−O(5)#2 | 148.3(6) |
| O(9)−Zn(3)−O(11)#1 | 89.6(4) | O(7)#3−Zn(6)−O(27) | 84.7(4) |
| O(9)−Zn(3)−O(17)#1 | 148.4(4) | O(7)#3−Zn(6)−O(28) | 70.7(4) |
| O(9)−Zn(3)−O(24) | 106.1(3) | O(20)−Zn(6)−O(5)#2 | 106.0(5) |
| O(9)−Zn(3)−O(29) | 86.4(7) | O(20)−Zn(6)−O(7)#3 | 105.2(4) |
| O(17)#1−Zn(3)−O(11)#1 | 87.2(3) | O(20)−Zn(6)−O(27) | 95.0(5) |
| O(17)#1−Zn(3)−O(29) | 79.0(5) | O(20)−Zn(6)−O(28) | 141.5(3) |
| O(24)−Zn(3)−O(11)#1 | 102.8(3) | O(23)−Zn(6)−O(5)#2 | 89.4(4) |
| O(24)−Zn(3)−O(17)#1 | 105.3(3) | O(23)−Zn(6)−O(7)#3 | 93.6(4) |
| O(24)−Zn(3)−O(29) | 111.0(9) | O(23)−Zn(6)−O(20) | 94.6(4) |
| O(29)−Zn(3)−O(11)#1 | 145.8(9) | O(23)−Zn(6)−O(27) | 170.3(6) |
| O(4)−Zn(4)−O(15) | 170.8(4) | O(23)−Zn(6)−O(28) | 49.0(3) |
| O(4)−Zn(4)−O(25) | 89.1(5) | O(27)−Zn(6)−O(28) | 121.8(5) |
| O(4)−Zn(4)−O(26) | 89.1(5) | O(2)#4−Zn(7)−O(23) | 96.5(4) |
| O(10)−Zn(4)−O(4) | 93.8(4) | O(2)#4−Zn(7)−O(30) | 81.2(5) |
| O(10)−Zn(4)−O(15) | 89.4(4) | O(8)#3−Zn(7)−O(2)#4 | 109.6(5) |
| O(10)−Zn(4)−O(25) | 83.5(4) | O(8)#3−Zn(7)−O(14) | 141.0(5) |
| O(10)−Zn(4)−O(26) | 168.9(4) | O(8)#3−Zn(7)−O(23) | 95.7(4) |
| O(15)−Zn(4)−O(25) | 85.5(5) | O(8)#3−Zn(7)−O(30) | 83.0(5) |
| O(15)−Zn(4)−O(26) | 86.1(5) | O(14)−Zn(7)−O(2)#4 | 106.5(4) |
| O(24)−Zn(4)−O(4) | 90.8(3) | O(14)−Zn(7)−O(23) | 94.5(3) |
| O(24)−Zn(4)−O(10) | 101.2(3) | O(14)−Zn(7)−O(30) | 88.5(5) |
| O(24)−Zn(4)−O(15) | 97.0(3) | O(23)−Zn(7)−O(30) | 176.6(5) |

Symmetry transformations used to generate equivalent atoms #1: –*y* + 1/3, *x* – *y* – 1/3, *z* – 1/3; #2: *x* + 1/3, *y* – 1/3, *z* – 1/3; #3: –*x* + *y* + 1, –*x*, *z*; #4: –*y* + 1, *x* – *y*, *z*.
